# Training Emotional Intelligence Online: An Evaluation of WEIT 2.0

**DOI:** 10.3390/jintelligence11060122

**Published:** 2023-06-15

**Authors:** Marco Jürgen Held, Theresa Fehn, Iris Katharina Gauglitz, Astrid Schütz

**Affiliations:** Institute for Psychology, University of Bamberg, 96047 Bamberg, Germany

**Keywords:** emotional intelligence, emotion perception, emotion regulation, online training, digital affinity

## Abstract

With the growing popularity of online courses, there is an increasing need for scientifically validated online interventions that can improve emotional competencies. We addressed this demand by evaluating an extended version of the Web-Based Emotional Intelligence Training (WEIT 2.0) program. Based on the four-branch model of emotional intelligence, WEIT 2.0 focuses on improving participants’ emotion perception and emotion regulation skills. A total of 214 participants were randomly assigned to the training group (*n* = 91) or a waiting list control group (*n* = 123) to evaluate short-term (directly after WEIT 2.0) and long-term intervention effects (8 weeks later). Two-way MANOVAs and mixed ANOVAs showed significant treatment effects for self-reported emotion perception of the self, as well as emotion regulation of the self and others, after 8 weeks. No significant treatment effects were found for self-reported emotion perception in others or for performance-based emotion perception or emotion regulation. Moderator analyses revealed no significant effects of digital affinity on training success from the pretest to the posttest. The findings suggest that components of self-reported emotional intelligence can be enhanced through WEIT 2.0, but performance-based emotional intelligence cannot. Further research is needed on the online training of emotional intelligence and the mechanisms that underlie training success.

## 1. Introduction

“I don’t want to be at the mercy of my emotions. I want to use them, to enjoy them, and to dominate them.”

—Oscar Wilde

Emotions, such as anger, sadness, disgust, or happiness, play an integral role in our lives. Long before the first official scientific definition, the Irish poet Oscar Wilde described the essence of what Salovey and Mayer (1990) would decades later call ability-related emotional intelligence (EI), namely, “the subset of social intelligence that involves the ability to monitor one’s own and others’ feelings and emotions, to discriminate among them and to use this information to guide one’s thinking and actions” (p. 189).

A plethora of studies have shown that the ability to master one‘s emotions is associated with better physical and mental health (Martins et al. 2010), higher quality of interpersonal relationships (Schröder-Abé and Schütz 2011), better job performance (Joseph et al. 2015), and higher job satisfaction (Miao et al. 2016). Given the numerous benefits of EI, different authors have made successful attempts to increase EI through face-to-face (F2F) training (Buruck et al. 2016; Herpertz et al. 2016; Hodzic et al. 2015). Despite the growing popularity of online courses (Gegenfurtner et al. 2020), only a few studies have examined whether the positive effects of F2F training can be generalized to the online setting (Köppe et al. 2019; Persich et al. 2021).

Various EI intervention studies have been criticized, as they were not theoretically grounded, focused on short-term changes rather than long-term ones, did not use performance-based measures of EI, and failed to randomly assign participants to experimental conditions (Schutte et al. 2013). In order to address such shortcomings, we based the extension of the Web-Based Emotional Intelligence Training (WEIT 2.0) program on the four-branch model of EI (Mayer and Salovey 1997) and randomly assigned participants to a control group (CG) or a training group (TG). In addition, we examined short-term and long-term changes in the individuals’ EI with the help of both self-report and performance-based measures. Finally, we explored the participants’ digital affinity as a potential skill that fostered the participants’ training success.

### 1.1. Ability-Related EI

In past decades, two distinct lines have emerged in the EI literature: (1) ability models; and (2) mixed or trait models (Mayer et al. 2008). In the mixed models, EI is viewed as an umbrella term that encompasses different personality traits, cognitive abilities, motivational constructs, interpersonal competencies, and emotional abilities (Bar-On 2006; Goleman 1995; Petrides and Furnham 2001). Various authors have criticized mixed models for including a wide variety of different constructs (e.g., Locke 2005), for rarely being based on a clear theoretical background (Mayer et al. 2008), and for having low discriminant and predictive validity (e.g., Joseph et al. 2015). 

On the basis of work by Thorndike (1920) and Gardner (1983), Salovey and Mayer (1990) introduced the four-branch model of EI, which distinguished four different facets: (1) emotion perception; (2) using emotions to facilitate thinking; (3) understanding emotions; and (4) emotion regulation. Introducing the four-branch model as a form of social intelligence, they focused the model on clearly defined abilities (Salovey and Mayer 1990). In addition, the authors assumed that the four branches developed across people’s lives and could be trained with the help of targeted interventions (Mayer and Salovey 1997). However, the four-branch model has come under criticism in recent years because several studies suggested that the second branch (using emotions) showed significant overlap with the other three branches (Joseph and Newman 2010; MacCann et al. 2014; Rossen et al. 2008). Thus, we did not address the second branch in our training program. Furthermore, the third branch, which focuses on the cognitive aspect of EI (understanding emotions), has also been criticized due to its overlap with verbal intelligence (Schütz and Koydemir 2018), and we, therefore, included limited training content on emotion knowledge. Overall, we provide participants with the basic emotional abilities of the four-branch model. Since the training content to improve emotion understanding in participants was quite limited, we did not evaluate participants’ development of their emotion knowledge and focused on improvements in emotion perception and regulation in the present study.

### 1.2. Relevance of Emotion Perception and Emotion Regulation

It is not surprising that emotion perception and emotion regulation are the most studied dimensions of the four-branch model, as they have shown significant associations with many important outcomes in practical settings (Herpertz et al. 2016). In the four-branch model of EI, emotion perception is the most basic facet and consists of the ability to recognize emotional states in the faces, voices, and behaviors of other individuals (emotion perception in others) as well as to accurately perceive one’s own emotions (emotion perception of the self) (Mayer and Salovey 1997). 

Individuals with better interpersonal emotion perception were found to report higher satisfaction with their interpersonal relationships, perform better at work, demonstrate more competence in social situations, and possess a wide range of positive personality traits (Hall et al. 2009). In line with these research results, low emotion perception skills have been associated with more depressive feelings, more somatic symptoms, and higher levels of stress (Robinson et al. 2012). In the work context, studies have demonstrated that the emotion perception of the self and others is negatively associated with burnout (Nizielski et al. 2013) and that the ability to accurately perceive other’s emotions is positively related to performance in jobs that include high emotional demands (Farh et al. 2012). In addition, salespeople who were more adept at reading others’ nonverbal emotional cues had higher increases in salaries and sales figures than their colleagues with poorer emotion perception skills (Byron et al. 2007).

The research results suggested that accurately perceiving the emotions of another individual is an important prerequisite for regulating the corresponding emotional state (Reeck et al. 2016). For instance, individuals with better emotion perception skills were found to be more sensitive in social interactions and to employ more adaptive strategies to regulate others’ emotions in interpersonal contexts (López-Pérez and Pacella 2021). Mayer and Salovey (1997) defined emotion regulation as the ability to select and apply the appropriate emotion regulation strategies to regulate both one’s own (emotion regulation of the self) and others’ emotional states (emotion regulation in others) to reach specific goals, making it the most complex facet of the four-branch model. 

Many patients who suffer from psychological disorders have exhibited significant deficits in regulating their own emotions (Berking and Wupperman 2012; Hertel et al. 2009). In addition, the ability to regulate emotions of the self and others has been found to significantly impact friendships, romantic relationships, and work relationships (Niven et al. 2015; Tamminen et al. 2019), as individuals with better interpersonal emotion regulation skills tend to be able to build trust in relationships (Niven et al. 2015). At the same time, dysfunctional intra- and interpersonal emotion regulations were found to be associated with an increase in conflicts (Lopes et al. 2011). As a result, the ability to regulate emotions in oneself and others has been associated with a higher quality of relationships (Lakey and Orehek 2011; Niven et al. 2012b) as well as higher subjective well-being in both interaction partners (Diamond and Aspinwall 2003; Niven et al. 2012a; Schröder-Abé and Schütz 2011). In the organizational world, people who have better overall emotion regulation skills and those who work in high-emotional labor jobs have been found to perform better at work (Joseph and Newman 2010). Finally, employees who have been good at managing emotions in themselves and others reported higher job satisfaction (Brackett et al. 2010). Given these benefits of both emotion perception and emotion regulation, our online course focused on improving these two key components of the four-branch model.

### 1.3. EI Interventions

Slaski and Cartwright (2003) were among the first to conduct a scientific study on an EI intervention and evaluate it with the managers. They found that only the TG, but not the CG, significantly improved their overall EI as well as their general health and psychological well-being. In 2008, Groves et al. (2008) demonstrated that the participants of an EI intervention, which was based on the four-branch model of EI (Mayer and Salovey 1997), showed improvements in all four emotional abilities. Since then, the research has gained substantial traction, with evaluation studies being conducted on many different target groups, such as students (e.g., Di Fabio and Kenny 2011; Viguer et al. 2017), teachers (e.g., Pérez-Escoda et al. 2012), employees (e.g., Buruck et al. 2016), athletes (e.g., Campo et al. 2019), and unemployed adults (e.g., Hodzic et al. 2015). Looking more closely at such EI interventions, however, it can be seen that they have varied greatly in duration as well as in the underlying theoretical models of EI they used. For instance, the duration varied from a few training days in the corporate setting (e.g., Slaski and Cartwright 2003) to two years in academic contexts (e.g., Viguer et al. 2017). Still, several studies showed that F2F training could improve participants’ EI and could have a positive impact on physical and mental health, the quality of social relationships, and life satisfaction (Kotsou et al. 2011; Nelis et al. 2009, 2011).

However, intervention studies on EI have remained subject to sustained criticism because they often displayed substantial methodological weaknesses (Geßler et al. 2021). Major shortcomings included the lack of an active CG and the failure to randomize participants to experimental conditions. Therefore, alternative explanations, such as placebo or Hawthorne effects, could not be ruled out (Shipstead et al. 2012). In addition, many studies did not use a theoretical model as the basis for their EI intervention (Zeidner et al. 2008), ignored long-term changes (Schutte et al. 2013), and lacked performance-based EI measures to explore training success (Köppe et al. 2019). 

Being aware of these limitations when choosing studies for their meta-analysis, Schutte et al. (2013) included studies (*k* = 4, *N* = 435) only if they based their EI intervention on a clear theoretical foundation, randomly assigned participants to experimental conditions, and measured participants’ EI at pretest and posttest with either a self-report or performance-based measure of EI. The authors found that participants’ EI increased as a result of the EI interventions. In 2018, Hodzic et al. conducted another meta-analysis (*k* = 28, *N* = 1986), using similar inclusion criteria but without insisting on the random assignment of participants to the experimental conditions. Consistent with Schutte et al.’s (2013) results, the authors reported that the EI interventions had a moderate effect on participants’ EI when comparing pretest and posttest results. An analysis of long-term effects showed that participants were able to retain the effects from the posttest to follow-up (Hodzic et al. 2018). In a recent meta-analysis, Mattingly and Kraiger (2019) examined the trainability of EI and included *k* = 14 studies (*N* = 582) that focused on ability-related EI interventions. They found that EI interventions had a moderate, positive effect on ability-related EI.

In the four-branch model of EI, EI is conceptualized as a set of emotional abilities (Mayer and Salovey 1997) that could be improved effectively through training (Hodzic et al. 2018; Schutte et al. 2013). Moderator analyses revealed that EI interventions that were based on ability models produced larger effect sizes compared with the interventions based on mixed models or no theoretical model (Hodzic et al. 2018). In addition, longer EI interventions proved superior to shorter EI interventions in terms of training success (Hodzic et al. 2018). Interestingly, later research results suggested that when emotion regulation and emotion perception were trained in conjunction, such an approach was more effective than when emotion perception was trained alone (Geßler et al. 2021). This finding supports our approach of integrating these two branches into one training program.

Even though F2F training has demonstrated positive effects on participants’ EI, and online interventions in positive psychology concepts are generally effective (Koydemir et al. 2021), there is still little research on the effectiveness of online EI interventions. Online interventions bring many benefits because they are more cost-effective; they can easily be accessed by a larger number of people, and they allow participants to learn at their own pace in a self-directed manner (Kimiloglu et al. 2017). Online interventions have been found to demonstrate success in other EI-related areas, such as positive psychology (Ouweneel et al. 2013), mindfulness (Spijkerman et al. 2016), and stress management (Hintz et al. 2015). Consequently, it is even more surprising that only a few studies have explored whether EI can be enhanced online.

Being one of the first online EI interventions, WEIT (Köppe et al. 2019) built on the four-branch model of EI (Mayer and Salovey 1997) and was designed to increase EI in future leaders. The online course consisted of four one-hour modules on emotion perception and emotion regulation, followed by a 4-week online follow-up. In their study, Köppe et al. (2019) used performance-based measures and a waiting list CG to assess training success. Results showed that the participants’ emotion perception skills improved directly after WEIT and remained stable 6 weeks afterward. Regarding emotion regulation, the TG showed improvements 6 weeks after WEIT. Interestingly, participants’ levels of stress were unaffected by the intervention. Another study by Persich et al. (2021) made use of an active CG (participation in awareness training) and employed self-report and performance-based EI measures to evaluate their online emotional intelligence training (EIT) program. Based on the four-branch model of EI (Mayer and Salovey 1997), the EIT program complemented the training content with other scientific, well-established emotion theories. By participating in the EIT program, participants were able to improve their emotion perception, emotion knowledge, and emotion regulation on both self-report and performance-based EI measures. Positive effects of EIT on EI were found even 6 months after the training program had ended. Taken together, these initial studies suggest that EI can also be enhanced in an online setting.

### 1.4. Self-Report vs. Performance-Based EI Measures

When assessing ability-based EI to measure training success, it is important to distinguish between self-report and performance-based measures. The two different measures seem to capture different aspects of EI, as research studies have reported low correlations between the two types of measurement (e.g., Brackett et al. 2006). Self-report measures tend to assess typical behavior rather than cognitive performance (Côté 2014), as they demonstrate stronger correlations with personality than with actual abilities (Mayer et al. 2008). By contrast, performance-based measures have been found to be more strongly related to cognitive abilities than to personality and allow researchers to compare respondents’ answers against a criterion of accuracy (Joseph and Newman 2010; Mayer et al. 2008). By using both self-report and performance-based measures of EI, we aim to capture different aspects of EI and counterbalance the advantages and disadvantages of the two measurement approaches (for an overview, see Côté 2014).

On the basis of research that has suggested that EI can be enhanced through F2F training (Hodzic et al. 2018; Schutte et al. 2013) and online interventions (Köppe et al. 2019; Persich et al. 2021), we posed the following hypotheses (see the preregistration):

**H1a.**  *Participants in the TG increase their self-reported and performance-based emotion perception and emotion regulation skills from the pretest to the posttest, whereas the scores of participants in the CG remain unchanged;*

**H1b.**  *Participants in the TG maintain their attained self-reported and performance-based emotion perception and emotion regulation skills from posttest to follow-up, whereas the scores of participants in the CG remain unchanged.*

### 1.5. Digital Affinity

Whether or not a training program is successful may depend, at least in part, on an individual’s personality (Herpertz et al. 2016). Research on traditional F2F training has shown that an individual’s personality influences their motivation to learn and to transfer such training and may, thus, enhance training effectiveness (e.g., Colquitt et al. 2000; Rowold 2007; Seeg et al. 2022). With regard to online training, it has also been proposed that an individual‘s characteristics could influence learning effectiveness (e.g., Arbaugh et al. 2009; Castro and Tumibay 2021). Thus, the participants in a training program may differ in how much they benefit from online training, for instance, depending on their levels of computer literacy or awareness and attitudes toward information and communication technology (Ali et al. 2018). However, until now, there has been little research on how participants’ personality influences their learning success in online interventions (Gegenfurtner et al. 2020; Kim and Schniederjans 2004). Given that an individual’s personality is crucial for training effectiveness in traditional F2F learning environments, we argue that it is vital to examine how personality characteristics influence the effectiveness of online training, such as our WEIT program. One personality variable that may be particularly relevant in this context is digital affinity.

Digital affinity is a personality trait that describes interindividual differences in the way people interact with digital interfaces (Franke et al. 2019). It is conceptualized as an individual’s approach/avoidance orientation toward an intensive interaction with technology (Franke et al. 2019). Thus, individuals with high digital affinity prefer to actively engage with technology, whereas individuals with low digital affinity prefer to avoid intensive interaction with technology (Franke et al. 2019). Accordingly, we assume that participants’ digital affinity may influence the extent to which participants approach or avoid the digital learning environment of our WEIT program. Digital affinity is an important personal resource that helps people cope successfully with technology (Franke et al. 2019). Participants who are high in digital affinity adapt more quickly and more successfully to new digital interfaces, such as online training, and show higher motivation to engage with such interfaces (Franke et al. 2019). For instance, Kim et al. (2019) showed that adaptation processes and attitudes toward the learning format were positively associated with learning success. We, therefore, propose the following (see the preregistration):

**H2.**  *Digital affinity moderates the success of training from pretest to posttest so that participants in the TG with the higher levels of digital affinity increase their self-reported and performance-based emotion perception and emotion regulation skills to a greater extent from pretest to posttest in comparison with the participants who have lower levels of digital affinity.*

## 2. Materials and Methods

### 2.1. Sample

**Figure 1:**
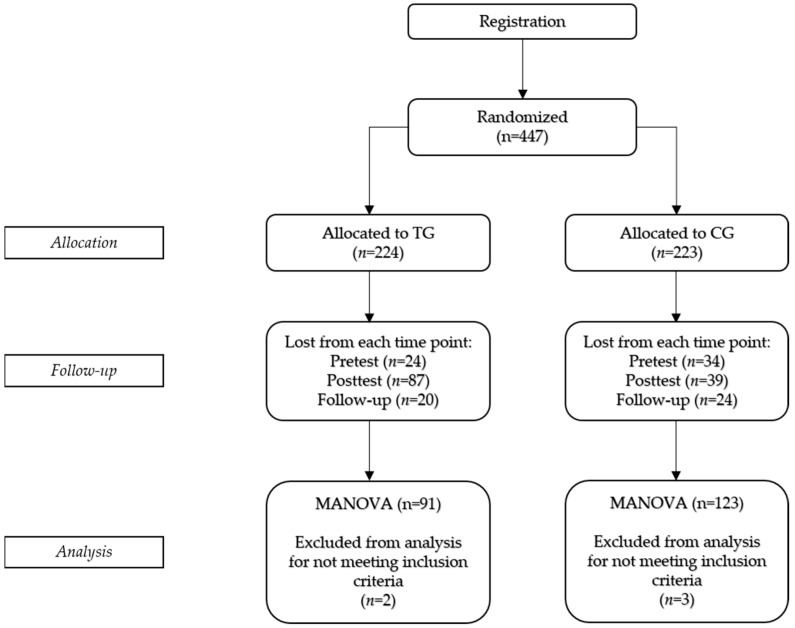
Participant flow diagram. TG = training group; CG = control group.

Participants were recruited through mailing lists, message posts, newsletters, newspapers, and contacts in corporate organizations. After signing up for the online intervention, 447 participants were randomly assigned to either the TG (*n_TG_* = 224) or the waiting list CG (*n_CG_* = 223). A total of 389 participants (*n_TG_* = 200, *n_CG_* = 189) completed the pretest; 263 participants (*n_TG_* = 113, *n_CG_* = 150) finished the posttest directly after the intervention, and 219 participants (*n_TG_* = 93, *n_CG_* = 126) filled out the follow-up 8 weeks after WEIT 2.0. Two cases (*n_TG_* = 1) were excluded because they completed the pretest, posttest, or follow-up in an unreasonably short amount of time. In addition, two participants from the CG were excluded due to extreme response behavior. Finally, one participant from the TG was excluded because the person was blind and, thus, unable to answer the Mayer Salovey Caruso Emotional Intelligence Test (MSCEIT; Mayer et al. 2002) items. As a result, the final sample consisted of 214 participants (*n_TG_* = 91, *n_CG_* = 123). Figure 1 presents the participant flow diagram for the study.

The mean age of the participants in the TG was *M* = 35.36 (*SD* = 14.61) years, with 70 participants identifying themselves as female (*n_male_* = 21). More than half of the participants in the TG (*n* = 83) stated that they had obtained at least a general higher education qualification as their highest degree. Regarding occupational status, the majority of the TG were students (*n* = 41) and employees (*n* = 31). The participants in the CG had an average age of *M* = 34.15 (*SD* = 13.73) and consisted of 89 female participants (*n_male_* = 33, *n_diverse_* = 1). Similar to the TG, the CG consisted of a large number of academically qualified individuals (*n* = 100) who had at least a general higher education qualification. Fifty-eight students and 48 employees were part of the CG, representing the two biggest groups in terms of occupation.

### 2.2. Procedure

The study was preregistered on OSF (https://osf.io/g43pz/ [accessed on 8 June 2023]).^1^ Participants were able to register until 4 November 2021 for the WEIT 2.0 program. Upon registration, participants were informed about two training cycles (October to December 2021, the TG; and January to February 2022, the CG) and asked in which week they would prefer to start if they were assigned to either cycle. Participants were then randomly assigned to one of the two training cycles (either the TG or the CG). Afterward, participants were provided with participant information regarding the online course and the three online surveys.

The training program was developed for the Virtuelle Hochschule Bayern (vhb), and the evaluation study was conducted within this setting. Unfortunately, no other online courses were available at the time of the study, which could have provided an active CG. We, therefore, decided to use a waitlist CG, though we are aware of the limitations of such a research design.

Data were collected online via SoSci Survey (https://www.soscisurvey.de/ [accessed on 8 June 2023]). Four days prior to the start of WEIT 2.0, the TG and CG were sent the link to the first online survey (pretest). After giving their consent, participants created a personalized code to match their data across the three measurement points. Next, we collected demographic data (i.e., gender, age, country of residence, educational status, employment status, and type of residence). Afterward, participants completed the subscales from the Self-Rated Emotional Intelligence Questionnaire (SREIS; Brackett et al. 2006; German version by Vöhringer et al. 2020), the Wong and Law Emotional Intelligence Scale (WLEIS; Wong and Law 2002), and the MSCEIT (Mayer et al. 2002; German version by Steinmayr et al. 2011). At the end of the pretest, participants filled out the ATI (Franke et al. 2019) and were asked to use their email addresses to register on the course platform (https://open.vhb.org/ [accessed on 8 June 2023]). 

Each training program started on a Monday. Participants in the TG received instructions on how to navigate the course and obtained an exemplary course schedule that recommended when to complete each chapter. Participants were given 3 weeks to complete the online course at their own pace and received automated reminders each week on Monday and Thursday. After the 3 weeks, participants were sent the link to the second online survey (posttest) and completed the SREIS (Vöhringer et al. 2020), the WLEIS (Wong and Law 2002), and the MSCEIT (Steinmayr et al. 2011). For further exploratory analyses, the TG also responded to items on the quality of the online course (e.g., structure, comprehensiveness) and whether they had completed each exercise.

Eight weeks later, we sent the link to the third online survey (follow-up). In this survey, participants again completed the SREIS (Vöhringer et al. 2020), the WLEIS (Wong and Law 2002), and the MSCEIT (Steinmayr et al. 2011). As an incentive to complete the follow-up, participants were given the options to obtain a training certificate, to be entered into a lottery for one of seven vouchers (1 × 100 Euro, 1 × 50 Euro, and 5 × 10 Euro), and to receive feedback on their EI, as measured with the MSCEIT (Steinmayr et al. 2011). At the end of each online survey, participants were asked to self-evaluate the quality of the data they had provided (“How thoroughly did you answer the survey?”) and whether they wanted to provide any comments. In addition, we used attention checks in each online survey to examine how conscientiously participants answered each of the three surveys. The CG answered the three online surveys parallel to the TG and started WEIT 2.0 after they completed the follow-up.

### 2.3. Web-Based Emotional Intelligence Training (WEIT 2.0)

The WEIT 2.0 program is a non-curricular, open online course that was offered through OPEN vhb (https://open.vhb.org/ [accessed on 8 June 2023]), a platform for open online courses developed by Bavarian universities that anyone can access free of charge after setting up a user account. The WEIT 2.0 program is an extension of the WEIT (Köppe et al. 2019) program. Both are based on the four-branch model of EI (Mayer and Salovey 1997) because interventions based on ability models of EI have shown greater effect sizes than interventions based on mixed models (Hodzic et al. 2018). Furthermore, past research has shown that EI interventions that are longer in duration have larger effects than shorter EI interventions. Therefore, WEIT 2.0 expanded the content in comparison with the initial version of WEIT (Köppe et al. 2019). In addition, WEIT 2.0 targeted the general population instead of future leaders. After teaching the fundamentals of EI, the online course focused on emotion perception and emotion regulation. The online course consisted of 13 modules, of which the first one was the introductory module and the last one was the concluding module. The remaining eleven modules covered the science of emotions in general (module 1), models and measurement of EI (modules 1 and 2), emotion knowledge (module 3), emotion perception of the self (modules 4 and 5), and others (modules 8 and 9), and emotion regulation of the self (modules 6 and 7) and others (modules 10 and 11). Table A1 (see Appendix A) displays the content of WEIT 2.0 in more detail.

The WEIT 2.0 program was developed on the basis of empirically sound theories and concepts in the field of EI. For example, we contrasted the theory of constructed emotions (Barrett 2017) with Paul Ekman’s (2005) theory of basic emotions to illustrate that the interpretation of contextual factors plays an important role in emotion perception beyond facial expression. Further, participants learned about stress appraisal theory (Lazarus and Folkman 1984) to understand that not only bodily sensations but also thoughts and appraisal processes are related to the onset of emotions. The modules on emotion regulation in oneself focused on different ways to downregulate negative emotions as well as to maintain and reinforce positive emotions. The process model of emotion regulation (Gross 1998) served as the theoretical basis of these modules. Finally, participants were introduced to important conflict and communication theories, such as the concept of nonviolent communication (Rosenberg 2015), to strengthen their interpersonal emotion regulation skills. 

We used a multimethod approach (e.g., learning videos, drag-and-drop exercises, quizzes, and audio files) and consistent feedback to teach EI. In the online course, participants were able to navigate freely through all modules and chapters. However, participants were advised to work on the training contents in the given order. They were able to contact the training team via email or an online forum when they encountered technical difficulties or when they had questions about the training contents. As we aimed to achieve long-term changes in participants, we designed the training program in accordance with the recommendations by Blume et al. (2010) and Seeg et al. (2022) to enhance training transfer. This is why we integrated elements, such as realistic training content, goal-setting exercises, and homework assignments, into the online course. Exploratory analyses revealed that it took participants an average of 60 to 90 min to complete each module, resulting in a total workload of approximately 18 h.

### 2.4. Measures

Self-reported EI was measured with the German version of the SREIS (Vöhringer et al. 2020). More specifically, we used the subscales Perceiving Emotion (SREIS-P), Managing Emotion (SREIS-M_1_), and Social Management (SREIS-M_2_), each of which contained four items. Participants rated how accurately each item described them on a 5-point Likert scale ranging from 1 (very inaccurate) to 5 (very accurate). Because the Perceiving Emotion subscale from the SREIS assessed only emotion perception in others (e.g., “By looking at people’s facial expressions, I recognize the emotions they are experiencing”), we additionally employed the Self-Emotions Appraisal (SEA) subscale from the WLEIS (Wong and Law 2002) to measure self-reported EI in the self. The WLEIS-SEA subscale contains four items that were rated on a 7-point Likert scale ranging from 1 (strongly disagree) to 7 (strongly agree). For the TG, Cronbach’s alpha ranged from .59 to .72 for the SREIS-P, from .75 to .82 for the SREIS-M_1_, from .71 to .76 for the SREIS-M_2_, and from .84 to .87 for the WLEIS-SEA. For the CG, Cronbach’s alpha varied from .64 to .68 for the SREIS-P, from .75 to .79 for the SREIS-M_1_, from .80 to .85 for the SREIS-M_2_, and from .86 to .89 for the WLEIS-SEA.

The German online version of the MSCEIT (Steinmayr et al. 2011) was used to measure performance-based EI. Emotion perception (MSCEIT-P) was assessed with the faces and images subtasks. In the faces subtask, participants are asked to use a 5-point scale to rate the degree to which each of the five emotions is expressed in a photograph. The images subtask is similar to the faces subtask, with the exception that landscapes and abstract patterns are displayed. Emotion regulation (MSCEIT-M) was measured with the emotion management and social management subtasks. Different situations are presented, and the effectiveness of strategies for attaining or maintaining a specific emotional state needs to be evaluated on a 5-point scale. While the emotion management subtask focuses on regulating emotions in the self, the social management subtask covers the regulation of emotions in others. Consensus scoring was used to calculate participants’ MSCEIT scores. For the TG, Cronbach’s alpha ranged from .86 to .91 across measurement points for the MSCEIT-P and from .46 to .61 for the MSCEIT-M. For the CG, Cronbach’s alpha varied from .87 to .89 for the MSCEIT-P and from .46 to .54 for the MSCEIT-M. Whereas the internal consistency of the MSCEIT-P was good, the internal consistency of the MSCEIT-M was not. This is in line with other studies examining the reliability of the MSCEIT and its respective subscales (Mayer et al. 2002). 

Finally, we assessed participants’ digital affinity with the Affinity for Technology Interaction (ATI; Franke et al. 2019) scale. The ATI scale encompasses nine items (e.g., “I like to occupy myself in greater detail with technical systems.”), which participants rated on a 6-point scale ranging from 1 (completely disagree) to 6 (completely agree). Higher ratings on the scale corresponded to higher digital affinity. Reliability analyses showed that Cronbach’s alpha was .93 in the TG and .92 in the CG.

### 2.5. Data Analysis

We analyzed the data with the software IBM SPSS Statistics Version 29. We ran two separate two-way MANOVAs for self-reported and performance-based EI with the within-subjects factor Time (Pretest, Posttest, Follow-up) and the between-subjects factor Group (Training Group, Control Group) to test for short-term (H1a) and long-term (H1b) intervention effects of the WEIT 2.0 program. In the case of a significant interaction, we followed up with mixed ANOVAs and examined simple main effects of group and time to investigate which patterns were responsible for the significant interaction. 

Before running the analyses, we checked whether all assumptions were met. We found neither univariate outliers nor multivariate outliers, as assessed with the Mahalanobis distance (*p* > .001). The assumption of multivariate normality was violated, as assessed with the Henze–Zirkler test statistic (*HZ* = 1.0064, *p* < .001). However, the parametric test statistic from a MANOVA is robust against the violation of the normality assumption and is superior to nonparametric test statistics with respect to power and the Type I error rate (Finch 2005). This is why we opted to use the parametric test statistic. Low to medium correlations (*r* < .90) between the dependent variables suggested that multicollinearity was not a major concern for the analysis. Finally, scatterplots challenged the assumption of linearity between self-reported and performance-based EI measures. As a result, we ran two separate MANOVAs, one for self-reported EI and one for performance-based EI.

We used a linear regression approach to test the moderating role of digital affinity on training success from the pretest to the posttest (H2). We employed MEMORE (Montoya 2019) to account for the fact that we used repeated-measures variables as predictors in our statistical model. In our analysis, we used bias-corrected bootstrapping with 5000 iterations to estimate 95 percent confidence intervals. MEMORE has the advantage that it can be used to probe significant interactions in a two-instance repeated-measures design by using either the pick-a-point approach or the Johnson–Neyman procedure.

## 3. Results

### 3.1. Descriptive Statistics

The training group had a mean digital affinity score of *M* = 3.39 (*SD* = 1.14) and a CG of *M* = 3.40 (*SD* = 1.06). Table 1 presents means and standard deviations for all outcome variables, separated by group and time point. 

The correlations of the self-reported EI measures with each other ranged from *r* = .26 to *r* = .54 (all *p*s < .001) at the pretest, from *r* = .25 to *r* =.50 (all *p*s < .001) at the posttest, and from *r* = .32 to *r* = .53 (all *p*s < .001) at the follow-up. The correlations of the performance-based EI measures with each other were .18 (*p* = .010) at the pretest, *r* = .10 (*p* = .142) at the posttest, and *r* = .19 (*p* = .006) at the follow-up. The correlations between the self-reported EI measures and the performance-based EI measures ranged from *r* = −.02 (*p* = .769) to *r* = .16 (*p* = .020) at the pretest, from *r* = −.04 (*p* = .546) to *r* = .12 (*p* = .081) at the posttest, and from *r* = −.07 (*p* = .330) to *r* = .20 (*p* = .004) at the follow-up. Table A2 (see Appendix A) contains the complete correlations for all measures at each measurement point. 

### 3.2. Self-Reported EI

Results from our first two-way MANOVA showed a statistically significant interaction between time and group (Wilk’s lambda Λ = .77, *F* [8, 205] = 7.57, *p* < .001). As the interaction was statistically significant, we next determined whether there were any statistically significant univariate interaction effects for each dependent variable. To do so, we first tested the assumption of sphericity for the repeated-measures variables using Mauchly’s test. Mauchly’s test was significant for the subscales Perceiving Emotion (SREIS-P; *p* < .001), Managing Emotion (SREIS-M_1_; *p* = .002), Social Management (SREIS-M_2_; *p* = .047), and Self-Emotions Appraisal (WLEIS-SEA; *p* < .001), meaning that the assumption of sphericity was violated for all self-reported EI scales. Therefore, we used the Greenhouse–Geiser adjustment to correct violations of sphericity. There was no statistically significant interaction between time and group for the SREIS-P (Greenhouse–Geisser *F* [1.79, 379.13] = 1.82, *p* = .167, *η*^2^ = .009), contradicting Hypotheses 1a and 1b to some extent. However, there was a statistically significant interaction between time and group for the SREIS-M_1_ (Greenhouse–Geisser *F* [1.89, 401.10] = 15.64, *p* < .001, *η*^2^ = .069), the SREIS-M_2_ (Greenhouse–Geisser *F* [1.95, 412.25] = 19.31, *p* < .001, *η*^2^ = .083), and the WLEIS-SEA (Greenhouse–Geisser *F* [1.86, 393.97] = 12.22, *p* < .001, *η*^2^ = .055).

In our follow-up mixed ANOVA for the SREIS-M_1_, we found no significant main effect of group (*F* [1, 212] = 3.25, *p* = .073, *η*^2^ = .015), but we did find a significant main effect of time (Wilk’s lambda Λ = .85, *F* [2, 211] = 18.36, *p* < .001, *η*^2^ = .148). Specifically, in the CG, there were no significant differences in the SREIS-M_1_ scores across time (Wilk’s lambda Λ = .97, *F* [2, 121] = 1.92, *p* = .151, *η*^2^ = .031), but in the TG, there were significant differences in the SREIS-M_1_ scores across time (Wilk’s lambda Λ = .72, *F* [2, 89] = 17.67, *p* < .001, *η*^2^ = .284). Participants in the TG had significantly higher SREIS-M_1_ values at the posttest compared with the pretest (−.34, *p* < .001), and their values remained unchanged from the posttest to the follow-up (−.02, *p* = 1.0). In sum, H1a and H1b were fully supported for the SREIS-M_1_.

Concerning the SREIS-M_2_, we found a significant main effect of group (*F* [1, 212] = 5.64, *p* = .018, *η*^2^ = .026) and a significant main effect of time (Wilk’s lambda Λ = .77, *F* [2, 211] = 31.14, *p* < .001, *η*^2^ = .228). Specifically, in the CG, there were no significant differences in the SREIS-M_2_ scores across time (Wilk’s lambda Λ = .97, *F* [2, 121] = 1.61, *p* = .203, *η*^2^ = .026). However, in the TG, there were significant differences in the SREIS-M_2_ scores across time (Wilk’s lambda Λ = .59, *F* [2, 89] = 31.16, *p* < .001, *η*^2^ = .412). Participants in the TG had significantly higher SREIS-M_1_ values at the posttest compared with the pretest (−.44, *p* < .001), and their values remained unchanged from the posttest to the follow-up (.04, *p* = 1.0). Altogether, H1a and H1b were fully supported for the SREIS-M_2_.

Results of our follow-up mixed ANOVAs for the WLEIS-SEA revealed no significant main effect of group (*F* [1, 212] = 1.08, *p* = .301, *η*^2^ = .005), but there was a significant main effect of time (Wilk’s lambda Λ = .86, *F* [2, 211] = 17.08, *p* < .001, *η*^2^ = .139). Specifically, in the CG, there were no significant differences in the WLEIS-SEA scores across time (Wilk’s lambda Λ = .99, *F* [2, 121] = 0.78, *p* = .462, *η*^2^ = .013), but in the TG, there were significant differences in the WLEIS-SEA scores across time (Wilk’s lambda Λ = .68, *F* [2, 89] = 20.98, *p* < .001, *η*^2^ = .320). Participants in the TG had significantly higher WLEIS-SEA values at the posttest compared with the pretest (−.42, *p* < .001), and their values remained unchanged from the posttest to the follow-up (−.06, *p* = 811). Thus, H1a and H1b were fully supported for the WLEIS-SEA.

### 3.3. Performance-Based EI

With regard to the performance-based EI, our second two-way MANOVA did not show a statistically significant Time x Group interaction effect (Wilk’s lambda Λ = .97, *F* [4, 209] = 1.39, *p* = .238). Therefore, Hypotheses 1a and 1b were not supported for the MSCEIT-P or for the MSCEIT-M.

**Figure 2:**
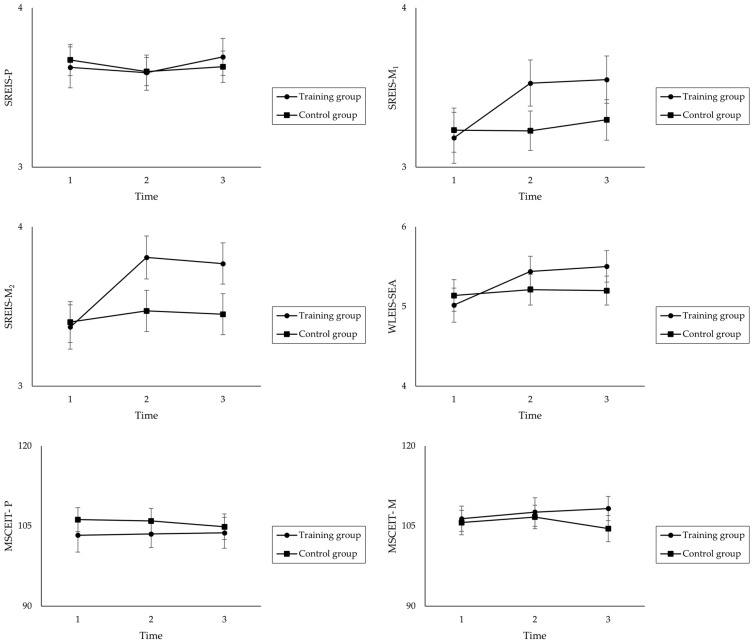
Graphs of interactions for all dependent variables across the pretest, posttest, and follow-up. Time 1 = pretest; Time 2 = posttest (directly after WEIT 2.0); Time 3 = follow-up (8 weeks later). SREIS-P = perceiving emotions in others; SREIS-M_1_ = managing emotions in the self; SREIS-M_2_ = managing emotions in others; WLEIS-SEA = appraising emotions in the self; MSCEIT-P = performance-based emotion perception; MSCEIT-M = performance-based emotion regulation.

Figure 2 shows the trajectories of each dependent variable from the pretest to the posttest to the follow-up.

### 3.4. Digital Affinity

Results of the multiple linear regression analyses with the MEMORE tool revealed that digital affinity did not moderate training success from pretest to posttest for the SREIS-P (*t* [89] = 0.16, *p* = .876), SREIS-M_1_ (*t* [89] = 0.84, *p* = .405), SREIS-M_2_ (*t* [89] = 0.07, *p* = .941), WLEIS-SEA (*t* [89] = −0.67, *p* = .502), MSCEIT-P (*t* [89] = −0.22, *p* = .823), or MSCEIT-M (*t* [89] = −0.12, *p* = .908). Therefore, Hypothesis 2 was not supported.

## 4. Discussion

Emotions play an essential role in people’s lives and permeate private as well as work lives. They allow people to enjoy their lives to the fullest and are important prerequisites for effective psychological functioning in society (Elfenbein et al. 2007; Hall et al. 2009). When emotion regulation is impaired, humans suffer, and emotional problems are part of many psychological disorders (Sheppes et al. 2015). Against this background, it is all the more important to be able to observe one’s own emotions and the emotions of others, to differentiate between them, and to use emotions to regulate one’s thinking and behavior—in short, to have EI (Salovey and Mayer 1990). Yet, not everyone possesses ability-related EI (Mayer et al. 2002), thus rendering it important to offer appropriate training. While F2F training has demonstrated success in improving individuals’ EI (Buruck et al. 2016; Herpertz et al. 2016; Hodzic et al. 2015), less is known about the effectiveness of online EI training (Köppe et al. 2019; Persich et al. 2021). In general, online training offers many advantages, such as flexibility in terms of when and where to participate, higher accessibility, or reduced costs, to name only a few (Kimiloglu et al. 2017). Accordingly, in order to train EI, it would be useful and advantageous to design such a training program as an online course. We carefully designed the WEIT 2.0 program built on a sound theoretical foundation (e.g., Barrett 2017; Gross 1998; Lazarus and Folkman 1984) and made use of recommendations for best practice (Blume et al. 2010; Seeg et al. 2022). In the following sections, we report on whether and to what extent WEIT 2.0 was effective and whether individual differences (i.e., in terms of digital affinity) had an impact on training effectiveness.

WEIT 2.0 is an open online course that focuses on improving individuals’ emotional competencies by building on the four-branch model of EI (Mayer and Salovey 1997). Participants were randomly assigned to either the TG or the waiting list CG and filled out measures on self-reported and performance-based EI at three measurement points (prior to WEIT 2.0 [pretest], directly after WEIT 2.0 [posttest], and 8 weeks later [follow-up]). We found that some facets of self-reported EI could be improved by WEIT 2.0, whereas performance-based EI remained unaffected by WEIT 2.0.

### 4.1. Theoretical Contributions

In a rapidly changing and digitalized world, learning virtually has become more important than ever, as it allows individuals to learn anytime and from anywhere (Kimiloglu et al. 2017). Another important advantage of online interventions is their cost-effectiveness because a very large number of participants can be trained, and the learning content can be personalized for each individual (Esteban-Millat et al. 2014). In the previous studies, online courses led to learning outcomes that were as good as, if not better, than F2F training (Sitzmann et al. 2006; Smith et al. 2015; Soffer and Nachmias 2018). With the onset of the COVID-19 pandemic, the need for online training has become greater than ever before. However, there is a lack of research on how participants will benefit the most from online training and how to best design a successful online intervention (Gegenfurtner et al. 2020). This is especially true in the field of EI, where only a few studies have probed whether EI can be improved through online training programs (e.g., Köppe et al. 2019; Persich et al. 2021).

Our research showed that the WEIT 2.0 program was an effective way to improve (in part) self-reported EI. Particularly, we found that through WEIT 2.0, individuals improved their self-rated abilities in managing emotions in the self and in others, as well as in appraising emotions in the self. By contrast, the ability to perceive emotions in others was not improved through WEIT 2.0. In line with previous research (Hodzic et al. 2018), training effects were still present even 8 weeks after training, meaning that WEIT 2.0 had long-term effects. This result shows that efforts to use a theoretically well-founded training concept with a multimethod approach in an online setting pay off at the individual level. We, thus, conclude that WEIT 2.0 is a successful adaptation and extension of WEIT (Köppe et al. 2019). In comparison with WEIT, which is targeted at leaders, WEIT 2.0 targets the general population, and, therefore, a larger group of people can access WEIT 2.0 and benefit from it.

Unexpectedly, and in contrast with previous research, which has shown that performance-based EI can be improved via training (Hodzic et al. 2018; Persich et al. 2021), in our study, performance-based EI was not improved through WEIT 2.0. One reason for this finding could be that WEIT 2.0 might not be ideally designed to improve ability-related EI as assessed by the MSCEIT. In addition, participants’ performance-based EI was already high before they participated in the training program, and it was, thereby, not easy to improve their EI further through training. Moreover, taking a look at the mean values of the performance-based EI scores at the pretest shows that the scores were already relatively high (with means ranging from 103.26 to 106.34) compared with the mean of ability-related EI in the general population, which usually has a value of 100 and an *SD* of 15 (Mayer et al. 2002). Exploratory analyses revealed that participants with higher performance-based EI at the pretest had a smaller increase in their performance-based EI than participants with lower performance-based EI at the pretest. This finding is in line with previous research that showed that individuals who demonstrated poorer EI skills were less likely to take part in EI training opportunities and were less receptive to negative feedback (Sheldon et al. 2014). By contrast, people with a well-developed skillset were more open to receiving further education (Sheldon et al. 2014). Thus, the lack of improvement may have also been due to a ceiling effect.

Unexpectedly, participants’ digital affinity did not influence training success. While it has been proposed that individual characteristics may influence training success in traditional F2F settings (e.g., Colquitt et al. 2000) but not in online settings (e.g., Arbaugh et al. 2009; Castro and Tumibay 2021), we could not find such an effect for WEIT 2.0, at least not for the individuals’ digital affinity. Self-selection could be a reason for this finding. As we advertised WEIT 2.0 as an online training program, it is possible that the individuals who agreed to participate may have been particularly open to such an online setting or, in terms of digital affinity, the people who volunteered may have had a high approach orientation with respect to digital environments. Yet, taking a look at the means of digital affinity in our sample, the TG participants scored lower in digital affinity than those in the standard sample in Franke et al. (2019). Another explanation could be that the online training program was well-designed, the program was not too complex, and the user interface was designed to be user-friendly so that all individuals, independent of their level of digital affinity, could profit from WEIT 2.0.

### 4.2. Limitations and Future Research

Our study has several limitations, which offer directions for future research. First, WEIT 2.0 was built on the four-branch model of EI and is focused on improving emotion perception and emotion regulation (Mayer and Salovey 1997). While we had a clear rationale for focusing on these two branches (e.g., as they are considered the two key EI-intervention components that are associated with the desired outcomes (Herpertz et al. 2016)), we do not know whether it is possible to train people to improve their skills in the other two branches, using emotions and understanding emotions. However, as the branches involving using emotions and understanding emotions have been criticized (e.g., with respect to the validity of these two branches (Joseph and Newman 2010; MacCann et al. 2014; Rossen et al. 2008)), we refrained from including them in WEIT 2.0. Future research could investigate whether and how using emotions and understanding emotions can be trained in an online setting.

Second, although we did not find support for our hypothesis that digital affinity would enhance training success, previous research has clearly indicated that individuals’ personal characteristics notably influenced training success (e.g., Arbaugh et al. 2009; Castro and Tumibay 2021). Therefore, we recommend that future research investigate other potentially relevant personal characteristics that may influence the training success of WEIT 2.0. For example, two individual characteristics that have been associated with training success are training motivation (Seeg et al. 2022) and conscientiousness (Kim and Schniederjans 2004). Future research could, therefore, address whether these individual characteristics can also influence the effectiveness of WEIT 2.0.

Third, due to the open accessibility of WEIT 2.0 and voluntary participation, the selectivity of participants may be an issue. Our sample consisted primarily of highly educated, young participants who already had high values on EI. Even though it is not surprising that well-educated people are especially likely to be open to participating in further training (Sheldon et al. 2014), we can draw conclusions about the effectiveness of WEIT 2.0 only for a population with similar characteristics (highly educated, young, emotionally intelligent). However, we do not know whether individuals who differ from our sample in these characteristics will also profit from WEIT 2.0 in a similar way. For instance, even though we tried to make our training program as understandable as possible, it remains open whether WEIT 2.0 is also comprehensible and useful to less educated people and will lead to similar training success. Furthermore, as younger people, in general, tend to interact more intensively with technology (Franke et al. 2019), it remains an open question whether older people will also profit from our online training program. Finally, we found that WEIT 2.0 improved self-reported EI in individuals with high initial values on EI. Regarding a population with lower EI, we would expect that WEIT 2.0 could be even more effective, as there would be more room for improvement. We, therefore, recommend evaluating WEIT 2.0 in a sample with less-educated, older, and less emotionally intelligent individuals. In order to achieve greater variability across participants, it would also be possible to offer the WEIT 2.0 course to a wider audience or to a group in an institution (e.g., in schools, higher education settings, or work settings).

Fourth, WEIT 2.0 was developed and tested in Germany, thus limiting the usability and range of its application. As we were able to demonstrate the effectiveness of the intervention in terms of self-rated EI, it would be conceivable to translate WEIT 2.0 into other languages and test its effectiveness. As cultural differences influence emotion perception and emotion regulation (Matsumoto and Wilson 2022), adaptions of WEIT 2.0 may also be necessary.

Fifth, whereas the emotion perception subscale from the MSCEIT showed good reliabilities in our study, the emotion regulation subscale did not. Although the reliabilities for this subscale are in agreement with the previous literature (Maul 2012), there is a need for a measure that can reliably assess emotion regulation. In future research, other ability-related EI measures could be used to investigate whether performance-based EI can be improved with WEIT 2.0.

Sixth, a disadvantage of self-report measures of EI is that they (1) can be affected by social desirability (Furnham 1986; Nederhof 1985) and (2) may reflect demand characteristics (Orne 1962). Future research could, therefore, control social desirability. Furthermore, we do not know whether self-reported increases in EI were associated with training transfer to participants’ daily lives (for example, if there was an impact on participants’ well-being or social relationships). Future research could examine whether WEIT 2.0 has such effects by including further measures of participants’ well-being or peer ratings indicating social relationship quality.

Finally, due to organizational issues, we were not able to implement an active CG. We, therefore, recommend that future research uses an active CG in order to make sure that improvements in the TG are not due to a placebo effect.

### 4.3. Practical Implications

The results of our study have several notable practical implications. First, the evaluation of WEIT 2.0 shows that online interventions are effective, at least in terms of improving self-reported EI. We speculate that training success can be traced back (at least in part) to a carefully designed training program. When designing WEIT 2.0, we grounded the training content on empirically sound theories (e.g., Barrett 2017; Gross 1998; Lazarus and Folkman 1984) and followed the recommendations for best practice (Blume et al. 2010; Seeg et al. 2022). As this approach appears to be feasible and efficient, we would like to encourage practitioners to develop future training content on a sound theoretical basis (e.g., the four-branch model of EI (Mayer and Salovey 1997)) and to follow the recommendations for best practice (e.g., by including elements, such as realistic training content, goal-setting exercises, and homework assignments) in future ability-related EI training programs. Moreover, in line with the previous research (Geßler et al. 2021), our study shows that emotion perception and emotion regulation can be effectively trained at the same time. Therefore, we recommend that practitioners also include both branches in one training program.

Furthermore, as longer EI interventions have been shown to have larger effect sizes than shorter EI interventions (Hodzic et al. 2018), we also recommend that practitioners develop future EI interventions with sufficient content mapping of all areas of EI that are of interest. For instance, WEIT 2.0 includes 13 modules that cover diverse aspects of EI based on two branches of the four-branch model of EI (Mayer and Salovey 1997).

For future EI training evaluations, we also recommend that researchers investigate not only the short-term effects (Schutte et al. 2013) but also the long-term effects of the intervention. By doing so, long-term training effectiveness can be evaluated, and researchers can determine whether training pays off in the long run. With regard to WEIT 2.0, long-term training effects were found for self-rated EI, as the effects were still present 8 weeks after training.

Finally, as EI is relevant to all areas of life, everyone can profit from a training program that is aimed at improving EI. This is why WEIT 2.0 was developed as an open online course that is accessible to anyone interested in this topic. We would, therefore, like to encourage practitioners to make future training available to the general population as well. As EI is associated with better health (Martins et al. 2010), higher interpersonal relationship qualities (Schröder-Abé and Schütz 2011), improved job performance (Joseph et al. 2015), and greater job satisfaction (Miao et al. 2016), open online courses could be beneficial for all members of various societies.

## Figures and Tables

**Table 1:** Means and standard deviations per measure, separated by group.

Outcome	Pretest				Posttest				Follow-Up			
	TG		CG		TG		CG		TG		CG	
	M	SD	M	SD	M	SD	M	SD	M	SD	M	SD
SREIS-P	3.62	0.62	3.67	0.55	3.59	0.53	3.60	0.50	3.69	0.56	3.63	0.56
SREIS-M_1_	3.18	0.81	3.23	0.75	3.53	0.68	3.23	0.71	3.55	0.73	3.30	0.71
SREIS-M_2_	3.37	0.67	3.40	0.72	3.81	0.65	3.47	0.73	3.77	0.62	3.45	0.72
WLEIS-SEA	5.2	1.03	5.14	1.11	5.44	0.91	5.21	1.08	5.50	0.95	5.20	1.02
MSCEIT-P	103.26	15.08	106.17	12.49	103.48	12.25	105.93	13.26	103.71	13.90	104.84	13.44
MSCEIT-M	106.34	11.36	105.63	12.73	107.59	12.85	106.67	12.39	108.26	10.88	104.50	13.72

Note. TG = control group; CG = control group; SREIS-P = perceiving emotions in others; SREIS-M_1_ = managing emotions in the self; SREIS-M_2_ = managing emotions in others; WLEIS-SEA = appraising emotions in the self; MSCEIT-P = performance-based emotion perception; MSCEIT-M = performance-based emotion regulation.

## Data Availability

The data presented in this study are openly available on OSF (https://osf.io/g43pz/ [accessed on 8 June 2023]).

## Note

1 Critical assumptions (i.e., measurement invariance) that were needed to test Hypotheses 1a and 1b using multigroup structural equation modeling were not met. Therefore, we deviated from our preregistration and employed two separate two-way MANOVAs to test for short- and long-term changes in self-reported and performance-based EI measures.

## Appendix A

**Table A1:** Content of WEIT 2.0.

Module		Content
1	Introduction to emotions and emotional intelligence	Definition, components, and functions of emotions Distinction between emotions, moods, and feelings Russell’s circumplex model of emotion Models of emotional intelligence (i.e., ability models, trait models, and mixed models)
2	Measurement of emotional intelligence	Importance of emotional intelligence in private life and work life Trainability of emotional intelligence Measurement approaches for the assessment of emotional intelligence Distinction between emotional intelligence, empathy, social competence and resilience
3	Emotion knowledge	Important emotion theories (e.g., theory of basic emotions, theory of constructed emotions) Enhancement of participants’ emotion vocabulary Internal and external triggers of emotions Temporal sequence and consequences of emotions
4	Emotion perception of the self (Part 1)	Introduction to emotion perception, emotion awareness and self-awareness Claude Steiner’s emotional literacy Bodily sensations and emotions (e.g., James-Lange theory, Schachter-Singer theory) Connection of facial expressions and gestures with emotions
5	Emotion perception of the self (Part 2)	Relation between cognitive processes and emotions Appraisal theories (e.g., Richard Lazarus, Magda Arnold) Reasoning errors and cognitive distortions
6	Emotion regulation of the self (Part 1)	Surface acting and deep acting Introduction to the process model of emotion regulation by James Gross Familiarization with different emotion regulation strategies (e.g., cognitive reappraisal, social support, suppression of emotions, relaxation methods, distraction, concentration)
7	Emotion regulation of the self (Part 2)	Application of different emotion regulation strategies to mitigate or intensify both pleasant and unpleasant emotions Reflecting on the effectiveness and appropriateness of learned emotion regulations strategies Development of an emotion plan for troubling emotions to better analyze one’s emotions
8	Emotion perception of others (Part 1)	Social and communicative functions of emotional expression Interpretation of different types of emotional and communicative signals (e.g., facial expression, body posture, voice) Emotion perception of others from facial expressions through images and videos
9	Emotion perception others (Part 2)	Cultural influences on the perception of emotions in others (e.g., display rules) Strategies for masking, intensifying and attenuating the expression of emotions Differences in the expression of emotions among different cultures
10	Emotion regulation in others (Part 1)	Communication and emotion regulation Theoretical fundamentals of traditional sender-receiver models Familiarization with different strategies of interpersonal emotion regulation (i.e., active listening)
11	Emotion regulation in others (Part 2)	Conflict management skills Introduction to nonviolent communication by Rosenberg Expressing appreciation and feedback towards others
12	Transfer into everyday life	Goal setting to enhance learning transfer with the help of SMART goals and implementation intentions

**Table A2:** Correlations of measures at pretest, posttest, and follow-up.

Variable		1	2	3	4	5	6
1	SREIS-P	1/1/1					
2	SREIS-M_1_	.26 ***/.25 ***/.32 ***	1/1/1				
3	SREIS-M_2_	.54 ***/.5 ***/.53 ***	.38 ***/.46 ***/.51 ***	1/1/1			
4	WLEIS-SEA	.37 ***/.37 ***/.4 ***	.33 ***/.44 ***/.41 ***	.34 ***/.37 ***/.40 ***	1/1/1		
5	MSCEIT-P	−.02/.01/0	.12/−.01/.05	.02/−.04/−.07	.09/.09/.13	1/1/1	
6	MSCEIT-M	.1/−.01/.13	.15 */.12/−0.02	.16 */.08/.1	.14 */.06/.2 **	.18 **/.10/.19 **	1/1/1
7	Digital Affinity	0/−.05/.01	.26 ***/.25 ***/.25 ***	.06/.06/.06	.08/.1/.07	−.01/.03/.08	−.01/−.01/−.13

Note. Correlations are presented separately according to time of measurement. Correlations at the pretest appear first; correlations at the posttest appear second, and correlations at the follow-up appear last. SREIS-P = perceiving emotions in others; SREIS-M_1_ = managing emotions in the self; SREIS-M_2_ = managing emotions in others; WLEIS-SEA = appraising emotions in the self; MSCEIT-P = performance-based emotion perception; MSCEIT-M = performance-based emotion regulation. * = *p* < .05, ** = *p* < .01, *** = *p* < .001.
